# Performance evaluation of PSO-PID and PSO-FLC for continuum robot’s developed modeling and control

**DOI:** 10.1038/s41598-023-50551-0

**Published:** 2024-01-06

**Authors:** Elsayed Atif Aner, Mohammed Ibrahim Awad, Omar M. Shehata

**Affiliations:** 1 Department of Mechatronics Engineering, Egyptian Russian University (ERU), Badr, 11829 Cairo Egypt; 2 Department of Mechatronics Engineering, Ain Shams University (ASU), Cairo, 11517 Cairo Egypt

**Keywords:** Biomedical engineering, Mechanical engineering

## Abstract

Continuum robots are complex structures that require sophisticated modeling and control methods to achieve accurate position and motion tracking along desired trajectories. They are highly coupled, nonlinear systems with multiple degrees of freedom that pose a significant challenge for conventional approaches. In this paper, we propose a system dynamic model based on the Euler–Lagrange formulation with the assumption of piecewise constant curvature (PCC), where we accounts for the elasticity and gravity effects of the continuum robot. We also develop and apply a particle swarm optimization (PSO) algorithm to optimize the parameters of our developed controllers: an inverse dynamic proportional integral derivative (PID) controller and an inverse dynamic fuzzy logic controller (FLC), where we use the integral time of absolute error (ITAE) as the objective function for the PSO algorithm. We validate our proposed model and optimized controllers through different designed trajectories, simulated using our developed unique animated MATLAB simulation. The results show that the PSO-PID controller improves the rise time, overshoot percentage, and settling time by 16.3%, 31.1%, and 64.9%, respectively, compared to the PID controller without PSO. The PSO-FLC controller shows the best performance among all controllers, with a settling time of 0.7 s and a rise time of 0.4 s, leading to the highest level of precision in trajectory tracking. The ITAE error for the PSO-FLC controller is 11.4% and 29.9% lower than that of the PSO-PID and FLC controllers, respectively.

## Introduction

Traditional robotic manipulators, which are generally Composed of a set of rotating joints and rigid linkages^1,2^, are widely used in industry. Rigid-link robots, however, pose a risk to delicate items and are unsuitable for interacting with humans. Robots that are capable of overcoming these restrictions and exhibit prominent levels of compliance and exceptional operational capabilities for environmental interaction and manipulating objects, known as soft continuum robots^3^, display a variety of innovative traits and have garnered a lot of interest.

Continuum manipulators belong to the category of soft robotics that are underactuated and often bio-inspired^4^. They mimic the natural motions of biological entities such as squid tentacles^5^, snakes^6^, and elephant trunks^7,8^.

Continuum robots (CRs) composed of a flexible backbone to which a number of discs are attached. Elastic deformation causes the structures of CRs to constantly curve along their length^9^. They can be constructed using numerous sections, giving them the potential to have an infinite degree of flexibility. Therefore, it is ideal for performing surgery with minimal invasion, medical uses^10^, and working in a complex and unstructured space^11^, where it can adapt to different shapes and handle objects and interact with the surroundings effectively.

Continuum robots are systemic complexity that is difficult to model and even more challenging to control, as they are a highly coupled-nonlinear system with limitless degrees of freedom^12^. Therefore derivation of accurate mathematical models is essential for the improved design, analysis, and control of continuum robots. Several methodologies and theories, including Denavit–Hartenberg parameters (DH)^13^, Euler–Bernoulli beam equation^14^, and Serret–Frenet frames^15^ have been exploited to construct a kinematic model, While a widely used assumption in the continuum robotics community is the PCC assumption^16^, which approximates the shape of the robot as a sequence of circular arcs that are tangent to each other, where a circular arc symbolizes the CR bending section, and the bending surface has the potential to rotate along a fixed axis. This assumption enables the simplification of the modeling of continuum robots. Various kinematic models based on the PCC assumption have been developed and applied successfully to different tasks, such as workspace analysis, trajectory tracking and whole-arm manipulation.

Finite element techniques^17^ and the Cosserat rod theory^18^ are excellent tools for describing system dynamics. Till et al.^19^ present a real-time simulation framework for soft and continuum robots based on Cosserat rod models. They derive the equations of motion using the principle of virtual work and discretize them using finite differences. F. Janabi-Sharifi^20^ provide a tutorial on Cosserat rod-based dynamic modeling of tendon-driven continuum robots. They review the basics of Cosserat rod theory and its application to continuum robots. It can be inferred that Cosserat rod theory provides a low-cost and flexible modeling approach for soft and continuum robots, which exhibit large shape deformations and environmental adaptability. However, this approach also faces some challenges, such as the need to simplify and approximate the robots’ physical properties and performance. It also requires precise knowledge of the material parameters and initial conditions, which may be difficult to obtain or estimate in practice.

Due to coupled, computationally costly formulae controlling the dynamics, the real-time application has only been feasible for simpler robots with a limited number of degrees of freedom. Therefore, it is difficult to use them in the construction of real-time dynamic controllers. The design of nonlinear resilient adaptive controllers is additionally challenging due to the structure of their dynamic equations.

In contrast, the Euler–Lagrange approach^21,22^, based on piecewise constant curvature assumption (PCC), uses system developed potential, and kinetic energy in developing the CR equation of motion. Where it considers system elasticity and gravity effect. Providing suitable inverse dynamics needed for model-based controller design or path planning.

Particle swarm optimization (PSO) is a popular meta-heuristic algorithm inspired by the collective behavior of social swarms in nature. It has been widely applied to various optimization problems in different domains, such as engineering, science, and business. However, PSO also faces some challenges, such as premature convergence, stagnation, and parameter tuning. To overcome these limitations, many variants, and modifications of PSO have been proposed^23^.

Again it is an extensive and time-consuming task to develop a reliable, efficient control algorithm for controlling the position and movement of soft continuum robotic manipulator along a desired trajectory.

Therefore, by proposing a two section CR dynamics model presented leveraging the Eular-Lagrange representation founded on the PCC assumption, we develop and apply two different control algorithms based on the utilized system inverse dynamics to handle system coupling and nonlinearity, (a) inverse dynamic PID controller. (b) inverse dynamics FLC. By this point, particle swarm optimization (PSO) was used as a tuning/optimization method developed and applied for both the PID and the FLC, where the PSO was used to optimize the parameter constants ${K}_{p}$, ${K}_{i}$ and ${K}_{d}$ of the developed PID, while for the developed FLC, the PSO was used to tune the membership function range for each input and output.

By the end, various dynamic control system outputs to a desired space parameter values were simulated and evaluated using MATLAB Simulink through designed trajectories (a) infinity shape. (b) rectangular path. Where the exported data is used in construction of a distinctive graphical interactively moving simulation.

The following points highlight the original contributions of this work:

- We proposed a two-section CR developed dynamic model using Eular-Lagrange representation founded on the assumption of the PCC, which accounts for its elasticity and gravity effect solved using a MATLAB symbolic toolbox.
- We Proposed two different control algorithms, inverse dynamic PID and inverse dynamic FLC that are carefully developed and implemented for accurate position trajectory tracking control, as they are further well optimized using developed multiple iteration PSO.
- The proposed PSO-optimized controllers are verified through the designed trajectory using MATLAB Simulink, where a comparative analysis is conducted to demonstrate the multiple dynamic responses and optimization enhancements for each designed controller in mapped two-dimensional trajectories, demonstrated using created unique animated simulation.

The rest of this work is structured as follows: Sect. “Kinematics modeling” introduces the robot’s mechanical structure and the kinematic model of the two-sections CR based on the PCC assumption, proceeded by the formulation of the dynamic model utilizing classical Euler–Lagrange representation, which is demonstrated in Sect. “Dynamics modeling”. PSO-based optimization of two control strategies and their development and analysis are discussed in Sect. “Controller design and optimization”. Section “Simulation and results” details the responses of the step and trajectory tracking, and how they are simulated with MATLAB and Simulink. At last, Sect. “Conclusion” concludes the paper and outlines further research plans.

## Kinematics modeling

**Figure 1:**
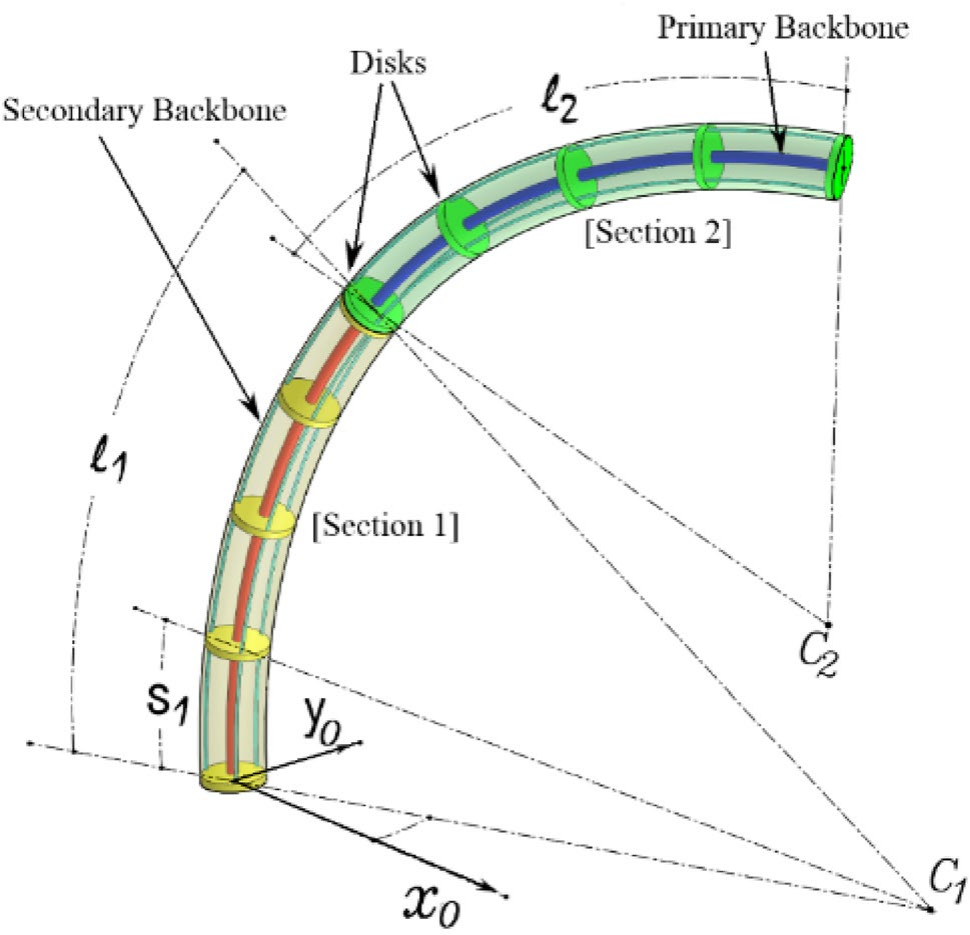
Design element for a two-section continuum robot.

The CR structure, demonstrated in Fig. 1 comprises a flexible backbone, three driving wires, and an array of disks. Each section core comprised of a long flexible backbone and five fixed disks that are evenly spaced. The disks have three circular holes that are 120° apart for the driving wires. The driving wires can exert a moment on the tip of the flexible backbone to manipulate the CR’s behavior. The CR’s spatial mobility hinges on the deflection of the flexible backbone, which is attained by applying suitable tension forces to one or two wires at a time. The CR undergoes a two-DOF bending motion, that can be utilized to construct a multi-degree of freedom continuum manipulator by utilizing multiple two degree of freedom links.

The continuum robot's modelling process begins with the development of a system kinematic model. Here, we first adopt the PCC assumption, which represents the configuration space of the CR as a limited number of mutually tangent curved segments, each of which has a constant curvature over its length.

**Figure 2:**
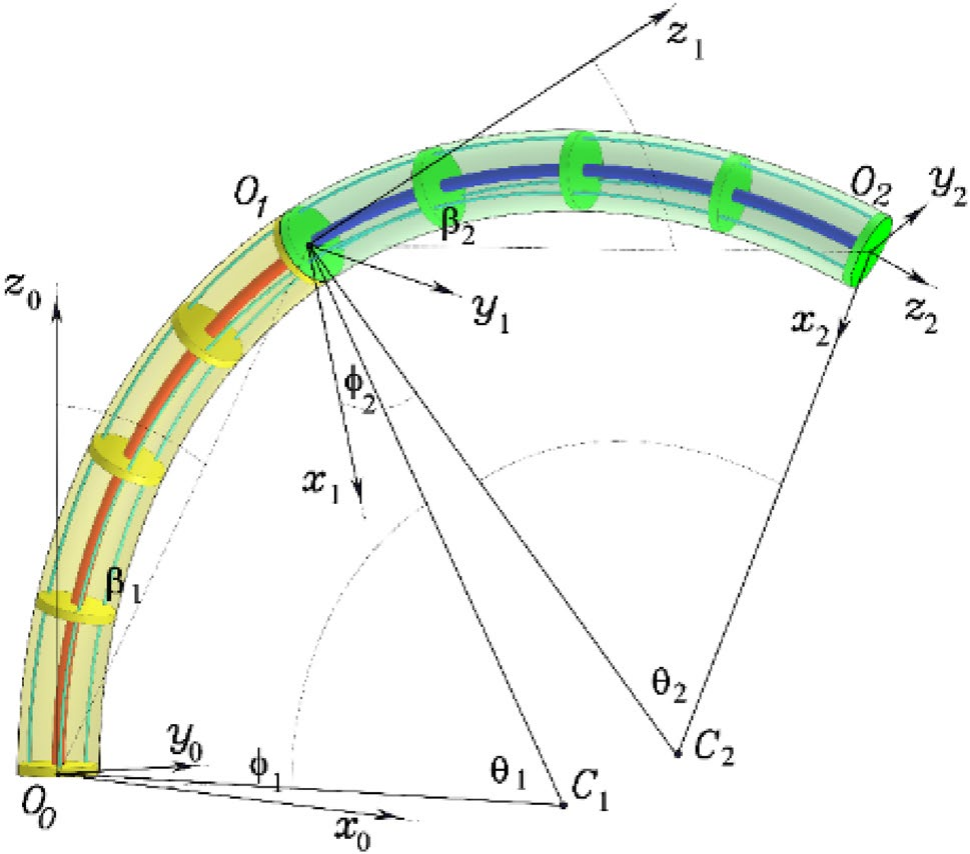
Parameters diagram for the two-section continuum robot.

The bending of each part of the CR is regarded as an arc based on the PCC assumption of modelling CRs given in Refs.^9,24^. Figure 2 shows the curvature parameters where, ${\theta }_{i}$ the angle that each section’s bending is represented by, ${\varphi }_{i}$ the arc plane corresponding angle, ${l}_{i}$ each section's arc length, ${r}_{i}$ radius of curvature, ${\beta }_{i}$ the tangent bending curve angle of the PB, ${s}_{i}$ the subsection’s arc length. Where $i$ represents section number.

1 $${\theta }_{{s}_{i}}={s}_{i}{\theta }_{i}/{l}_{i}.$$

Equation (1) represent subsection bending angle ${\theta }_{{s}_{i}}$, while the robot configuration space corresponds to ${\text{q}}\in {{\text{R}}}^{4}$, where ${\text{q}}={\left[{\theta }_{1}{\varphi }_{1}{\theta }_{2}{\varphi }_{2}\right]}^{{\text{T}}}$ as demonstrated in Fig. 2.

### Forward kinematics

Each ith section’s transformation matrix in regard to the [frame $i-1$] expressed by ${{\text{T}}}_{i}^{i-1}\in {{\text{R}}}^{4\times 4}$ as follows^9,25^:

2 $${\mathbf{T}}_{i}^{i-1}=\left[\begin{array}{cccc}{\text{cos}}{\varphi }_{i}{\text{cos}}{\theta }_{i}& -{\text{sin}}{\varphi }_{i}& {\text{cos}}{\varphi }_{i}{\text{sin}}{\theta }_{i}& {r}_{i}{\text{cos}}{\varphi }_{i}\left(1-{\text{cos}}{\theta }_{i}\right)\\ {\text{sin}}{\varphi }_{i}{\text{cos}}{\theta }_{i}& {\text{cos}}{\varphi }_{i}& {\text{sin}}{\varphi }_{i}{\text{sin}}{\theta }_{i}& {r}_{i}{\text{sin}}{\varphi }_{i}\left(1-{\text{cos}}{\theta }_{i}\right)\\ -{\text{sin}}{\theta }_{i}& 0& {\text{cos}}{\theta }_{i}& {r}_{i}{\text{sin}}{\theta }_{i}\\ 0& 0& 0& 1\end{array}\right].$$

The subsequent matrix denotes the final location of each subsection relative to the [frame $i-1$], given ${r}_{i}= {l}_{i}/{\theta }_{i}$.

3 $${{\text{O}}}_{{s}_{i}}^{i-1}={\left[{O}_{{s}_{i(x)}}^{i-1}{O}_{{s}_{i(y)}}^{i-1}{O}_{{s}_{i(z)}}^{i-1}\right]}^{T}=\left[\begin{array}{c}{r}_{i}{\text{cos}}{\varphi }_{i}\left(1-{\text{cos}}{\theta }_{{s}_{i}}\right)\\ {r}_{i}{\text{sin}}{\varphi }_{i}\left(1-{\text{cos}}{\theta }_{{s}_{i}}\right)\\ {r}_{i}{\text{sin}}{\theta }_{{s}_{i}}\end{array}\right].$$

The position of each section’s end relative to [frame 0], ${0}_{1}^{0}$ and ${0}_{2}^{0}$ are obtained by substituting $i=1, 2$ and ${s}_{1}={l}_{1}, {l}_{2}$, respectively. Where the position of subsection in second section ${{\text{O}}}_{{s}_{2}}^{0}$ expressed as:

4 $$\left[\begin{array}{c}{{\text{O}}}_{{s}_{2}}^{0}\\ 1\end{array}\right]={\left[\begin{array}{llll}{O}_{{s}_{2\left(x\right)}}^{0}& {O}_{{s}_{2\left(y\right)}}^{0}& {O}_{{s}_{2\left(z\right)}}^{0}& 1\end{array}\right]}^{T}={{\text{T}}}_{1}^{0}\left[\begin{array}{c}{{\text{O}}}_{{s}_{2}}^{1}\\ 1\end{array}\right].$$

where the second section end position (end effector) with regard to [Frame 0] $\left({0}_{2}^{0}\right)$ are obtained by simply substituting ${s}_{2}={l}_{2}$ in (4).

### Inverse kinematics

Given the terminal locations of each segment relative to [frame 0] depicted in Fig. 2, the configuration angles $\left[{\theta }_{1} {\varphi }_{1} {\beta }_{1} {\theta }_{2} {\varphi }_{2} {\beta }_{2}\right]$ of the two-sections CR can be derived geometrically as follows^9^:

5 $${\varphi }_{i}={{\text{tan}}}^{-1}\left({O}_{{i}_{\left(y\right)}}^{i-1}/{O}_{{i}_{\left(x\right)}}^{i-1}\right),$$

6 $${\beta }_{i}={{\text{tan}}}^{-1}\left(\sqrt{{\left[{O}_{{i}_{\left(x\right)}}^{i-1}\right]}^{2}+{\left[{O}_{{i}_{\left(y\right)}}^{i-1}\right]}^{2}}/{O}_{{i}_{\left(z\right)}}^{i-1}\right),$$

7 $${\theta }_{i}=\left\{\begin{array}{c}2{\beta }_{i}, \,\,\,\,\,\,\,\,\,\,\,\,\,{ O}_{{i}_{\left(z\right)}}^{i-1}>0\\ \pi , \,\,\,\,\,\,\,\,\,\,\,\,\,\,\,\,\,\,{O}_{{i}_{\left(z\right)}}^{i-1}=0\\ 2\pi +2{\beta }_{i}, \,\,\,\,\,\,{ O}_{{i}_{\left(z\right)}}^{i-1}<0\end{array}\right.$$

## Dynamics modeling

As demonstrated in Fig. 1, continuum robot consists of segments, each of which comprises of a primary backbone (PB), parallel secondary backbones (SBs) distributed 120 degrees apart, and multiple discs.

This section presents dynamic model of a two-section CR using Eular-Lagrange representation, through which kinetic and detailed-potential energy of each subsection are presented. In addition, symbolic-math toolbox of (MATLAB) was used to solve system partial differential equation.

### Kinetic energy derivation

Kinetic energy is consisting of three component of the three parts PB, SBs, and discs for each section. Firstly, the kinetic energy component belonging to the PBs denoted by $K{E}_{Pb}$ attainable as follows:

8 $$K{E}_{Pb}=\frac{1}{2}\sum_{i=1}^{2} {\int }_{0}^{{l}_{i}} \left[{\left(\frac{d{O}_{{s}_{i}\left(x\right)}^{0}}{dt}\right)}^{2}+{\left(\frac{d{O}_{{s}_{i}\left(y\right)}^{0}}{dt}\right)}^{2}+{\left(\frac{d{O}_{{s}_{i}\left(z\right)}^{0}}{dt}\right)}^{2}\right]{\rho }_{P}{A}_{P}d{s}_{i}+ \frac{1}{2}\sum_{i=1}^{2} {\int }_{0}^{{l}_{i}} \left[{\left(\frac{{s}_{i}{\dot{\theta }}_{i}}{{l}_{i}}\right)}^{2}+{\left({\dot{\varphi }}_{i}\right)}^{2}\right]{\rho }_{P}{I}_{P}d{s}_{i},$$

where, ${\rho }_{P}$ denotes the density, ${A}_{P}$ represents cross-section area, and ${I}_{P}$ signifies the PB second moment of cross-sectional area.

The following method is used to determine the SB lengths:

9 $$\begin{array}{l}{l}_{i,1}={l}_{i}-\alpha {\theta }_{i}{cos}\left(\sum_{k=1}^{i} {\varphi }_{k}\right),\\ {l}_{i,2}={l}_{i}-\alpha {\theta }_{i}{cos}\left({120}^{\circ }-\sum_{k=1}^{i} {\varphi }_{k}\right), \\ {l}_{i,3}={l}_{i}-\alpha {\theta }_{i}{cos}\left({120}^{\circ }+\sum_{k=1}^{i} {\varphi }_{k}\right),\end{array}$$

where, $\alpha$ is the gap between the primary backbone and each secondary backbone.

The system kinetic energy of the SBs is the second component, it is separated into two parts. By replacing each $\frac{1}{2}$ with $\frac{3}{2}$ and each subscript "P" insisted of "S" in (8), The first component $\left(K{E}_{S{b}_{1}}\right)$ can be computed.

The second component can be built as follows by differentiating the actuators' lengths:

10 $${{\text{KE}}}_{{{\text{Sb}}}_{2}}=\frac{1}{2}\sum_{{\text{i}}=1}^{2} {{\text{m}}}_{{\text{S}}}\left[{\left(\frac{{{\text{dl}}}_{{\text{i}},1}}{{\text{dt}}}\right)}^{2}+{\left(\frac{{{\text{dl}}}_{{\text{i}},2}}{{\text{dt}}}\right)}^{2}+{\left(\frac{{{\text{dl}}}_{{\text{i}},3}}{{\text{dt}}}\right)}^{2}\right],$$

where, ${m}_{S}$ is the mass of each ${\text{SB}}$.

The third component of the kinetic energy, is the discs kinetic energy $K{E}_{D}$, which can be obtained by deriving the velocity of each disc. By substituting ${s}_{i}={k}_{i}h$ in ${{\text{O}}}_{{s}_{i}}^{0}$ the position of each disc ${{\text{O}}}_{{d}_{i}}^{0}$ is obtained. Where $K{E}_{D}$ can be articulated by:

11 $$K{E}_{D}={\sum }_{i=1}^{2} \left[\frac{1}{2}{\sum }_{{k}_{i}=1}^{{n}_{i}} {m}_{D}\left[{\left(\frac{d{O}_{{d}_{i}(x)}^{0}}{dt}\right)}^{2}+{\left(\frac{d{O}_{{d}_{i}(y)}^{0}}{dt}\right)}^{2}\right.\right.\left.\left.+{\left(\frac{d{O}_{{d}_{i}(z)}^{0}}{dt}\right)}^{2}\right]+\frac{1}{2}{\sum }_{{k}_{i}=1}^{{n}_{i}} {I}_{D}\left[{\left(\frac{{k}_{i}h{\dot{\theta }}_{i}}{{l}_{i}}\right)}^{2}+{\left({\dot{\varphi }}_{i}\right)}^{2}\right]\right].$$

The letters ${n}_{1}$ and ${n}_{2}$ denote the disc count in the initial and subsequent segments of the CR, respectively. Where $h$ represents the space between each disc. and ${k}_{i}=\mathrm{1,2},3,\dots ,{n}_{i}$. The symbols ${m}_{D}$ and ${I}_{D}$ represent every disk mass and the mass moment of inertia.

Employing Eqs. (8), (10) and (11) the total kinetic energy *KE* is denoted by:

12 $$KE=K{E}_{Pb}+K{E}_{S{b}_{1}}+K{E}_{S{b}_{2}}+K{E}_{D}.$$

### Potential energy derivation

The CR has two types of potential energy: the potential energy due to gravity and elasticity. These are derived from both the PB and the discs.

Given that the orientation of the positive ${x}_{0}-{\text{axis}}$ coincides with the gravitational acceleration (g). The formula for the gravitational potential energy component of the PB $\left(GP{E}_{Pb}\right)$ is given by:

13 $$GP{E}_{Pb}=\sum_{i=1}^{2} {\int }_{0}^{{l}_{i}} \left[{O}_{{s}_{i}\left(x\right)}^{0}\right]{\rho }_{P}{A}_{P}gd{s}_{i}.$$

The total gravitational potential energy of the two-sections continuum robot $GP{E}_{D}$ denoted as following:

14 $$GP{E}_{D}=\sum_{i=1}^{2} \left[\sum_{{k}_{i}=1}^{{n}_{i}} {m}_{D}g{O}_{{d}_{i}\left(x\right)}^{0}\right].$$

The elastic potential energy is provided by:

15 $$\begin{array}{cc}EPE& =\sum_{i=1}^{2} \left[{\int }_{0}^{{l}_{i}} \frac{{E}_{P}{I}_{P}}{2}{\left(\frac{d{\theta }_{{s}_{i}}}{d{s}_{i}}\right)}^{2}d{s}_{i}+3{\int }_{0}^{{l}_{i}} \frac{{E}_{S}{I}_{S}}{2}{\left(\frac{d{\theta }_{{s}_{i}}}{d{s}_{i}}\right)}^{2}d{s}_{i}\right]\\ & \end{array} = \frac{{E}_{P}{I}_{P}}{2{l}_{1}}{\theta }_{1}^{2}+\frac{3}{2}\frac{{E}_{S}{I}_{S}}{{l}_{1}}{\theta }_{1}^{2}+\frac{{E}_{P}{I}_{P}}{2{l}_{2}}{\theta }_{2}^{2}+\frac{3}{2}\frac{{E}_{S}{I}_{S}}{{l}_{2}}{\theta }_{2}^{2}.$$

where, ${E}_{P}$ and ${E}_{S}$ denotes $PB$ and SBs elasticity modules, respectively.

By summing all the potential energy components (13), (14) and (15) system potential energy $(PE)$ can be obtained.

16 $$PE= GP{E}_{Pb}+GP{E}_{D}+EPE.$$

### Equation of motion

Using differentials of Eqs. (12) and (16) we can form the Lagrange equation^21,22,25^:

17 $$\frac{d}{dt}\frac{\partial KE}{\partial {\dot{q}}_{\left(j\right)}}-\frac{\partial KE}{\partial {q}_{\left(j\right)}}+\frac{\partial PE}{\partial {q}_{\left(j\right)}}={\tau }_{\left(j\right),}$$

where $q=\left[{\theta }_{1}{\varphi }_{1}{\theta }_{2}{\varphi }_{2}\right]$ represent configurations space variable, and $\dot{q}=\left[{\dot{\theta }}_{1}{\dot{\varphi }}_{1}{\dot{\theta }}_{2}{\dot{\varphi }}_{2}\right]$ represent its derivatives.

The equation of motion can be expressed in its final form as follows:

18 $${\text{M}}\left({\text{q}}\right)\ddot{{\text{q}}}+{\text{V}}\left({\text{q}},\dot{{\text{q}}}\right)\dot{{\text{q}}}+{\text{G}}\left({\text{q}}\right)=\tau ,$$

where, ${\text{M}}\left({\text{q}}\right)$ presents the inertia matrix of dimension 4 × 4 $,{\text{V}}\left({\text{q}}\right)$ is the 4 × 4 matrix that contains the centrifugal Coriolis torque components $,{\text{G}}\left({\text{q}}\right)$ is the 4-dimensional vector that represents the gravitational torques while $\tau$ is the 4-dimensional vector of the CR torques.

## Controller design and optimization

Controlling continuum robot arms poses significant challenges due to their highly nonlinear and coupled dynamics. In this section, we aim to develop and apply two different controllers whose parameters are optimized using particle swarm optimization. There are inverse dynamic PID and inverse dynamic FLC.

The system configuration space error ${\text{e}}\left(t\right)\in {\mathbb{R}}^{4}$ (error vector), defined as:

19 $${\text{e}}\left(t\right)={{\text{q}}}_{\left(d\right)}\left(t\right)-{\text{q}}\left(t\right)={\left[{e}_{\left({\theta }_{1}\right)}{e}_{\left({\varphi }_{1}\right)}{e}_{\left({\theta }_{2}\right)}{e}_{\left({\varphi }_{2}\right)}\right]}^{T}$$

where the desire configuration space angle denoted as ${q}_{\left(d\right)}={\left[{\theta }_{{1}_{\left(d\right)}}{\varphi }_{{1}_{\left(d\right)}}{\theta }_{{2}_{\left(d\right)}}{\varphi }_{{2}_{\left(d\right)}}\right]}^{T}$, which, in the instance of trajectory tracing, could be a time function.

### PSO optimized PID controller

The objective is to ensure that the CR time parameters coordinates *q*(*t*) converge to a desired time profile defined by the vector ***qd***(*t*), such that the error ***e***(***t***) = ***qd***(***t***) − ***q***(***t***) vanishes asymptotically. A second-order error equation is required for this purpose. A linear form of the error equation can be selected as follows:

20 $$\ddot{e}+{K}_{d}\dot{e}+{K}_{p}e+ {K}_{i}\int e \,dt=0.$$

The coordinate vector q’s second-order time derivative can be obtained from (20) as:

21 $$\ddot{q}=\ddot{{q}_{d}}+{K}_{d}\left(\dot{{q}_{d}}-\dot{q}\right)+{K}_{p}\left({q}_{d}-q\right)+{K}_{i}\int \left({q}_{d}-q\right) dt.$$

The purpose of our controller is to choose a suitable control input ${\text{U}}\left(t\right)\in {\mathbb{R}}^{4}$ to converge the tracking error ${\text{e}}\left(t\right)$ to zero. We derive the following inverse dynamic control law: The resulting control law is obtained by inserting Eq. (21) into the system dynamic model (18):

22 $${\text{M}}\left({\text{q}}\right)\left({\ddot{q}}_{d}+{K}_{p}e+ {K}_{i}\int e \,dt+{K}_{d}\dot{e}\right)+\left[{\text{V}}\left({\text{q}},\dot{{\text{q}}}\right)\dot{{\text{q}}}+{\text{G}}\left({\text{q}}\right)\right]={t}_{PID}.$$

where ${K}_{p}$, ${K}_{i}$ and ${K}_{d}$ are the proportional, integral constant control gains, respectively. ${\ddot{q}}_{d}\in {\mathbb{R}}^{4}$ is the second derivative value of the desired trajectory. By choosing a set of appropriate gain values of ${K}_{p}$, ${K}_{i}$ and ${K}_{d}$ the configuration variables of the close-loop system is able to track the desired trajectory and minimize the error e(t).

**Figure 3:**
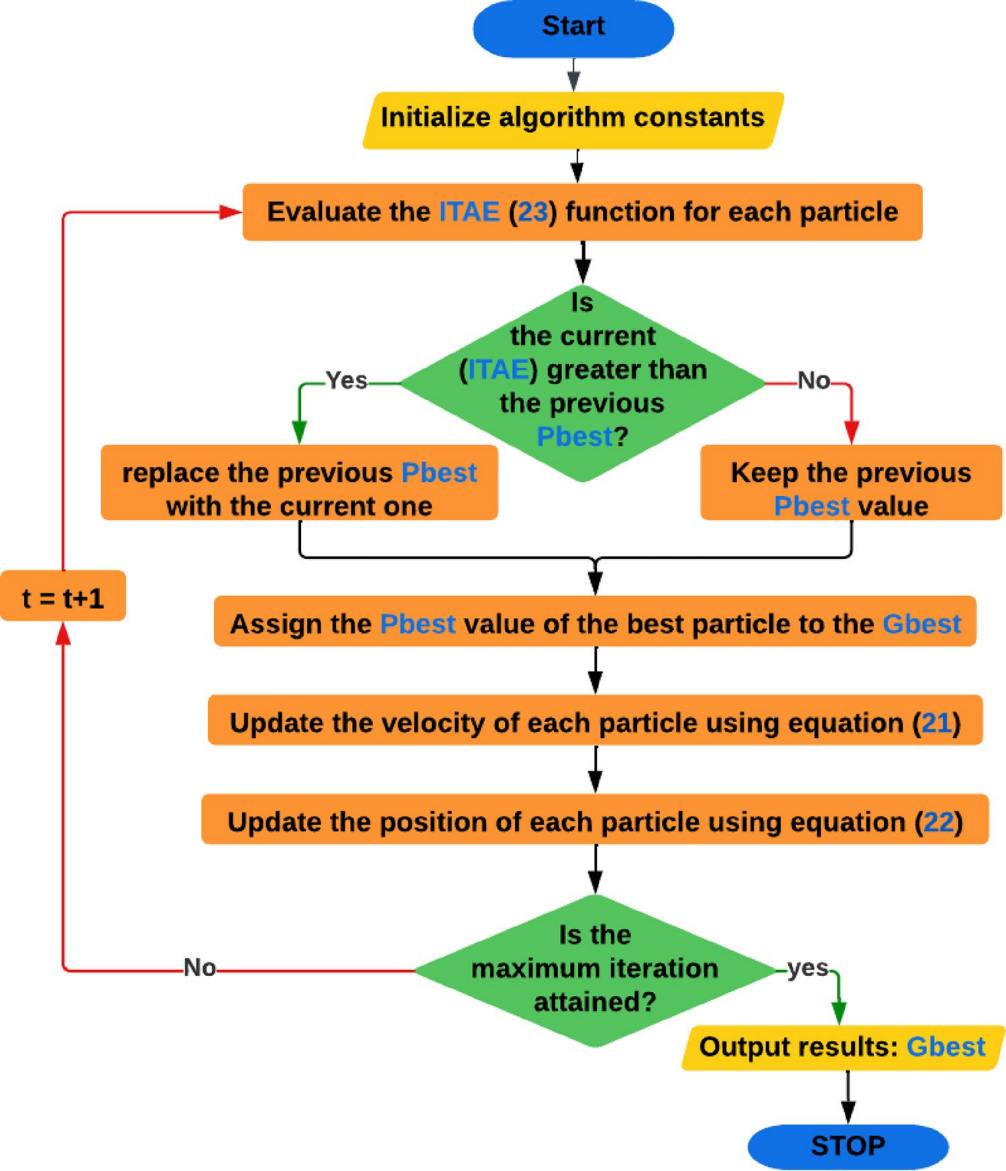
Developed PSO flowchart.

PSO algorithm is a swarm-based search method that operates on a D-dimensional solution space. Each potential solution is represented by a particle that has a position and a velocity and can store the best position of itself and the swarm. At each iteration, the particles update their velocities and positions based on the information of their best positions. The particles dynamically explore the solution space until they converge to an optimal or near optimal state or exceed the computational budget. The objective functions provide the linkage among different dimensions of the problem space. Figure 3 demonstrate our PSO algorithm flowchart.

**Figure 4:**
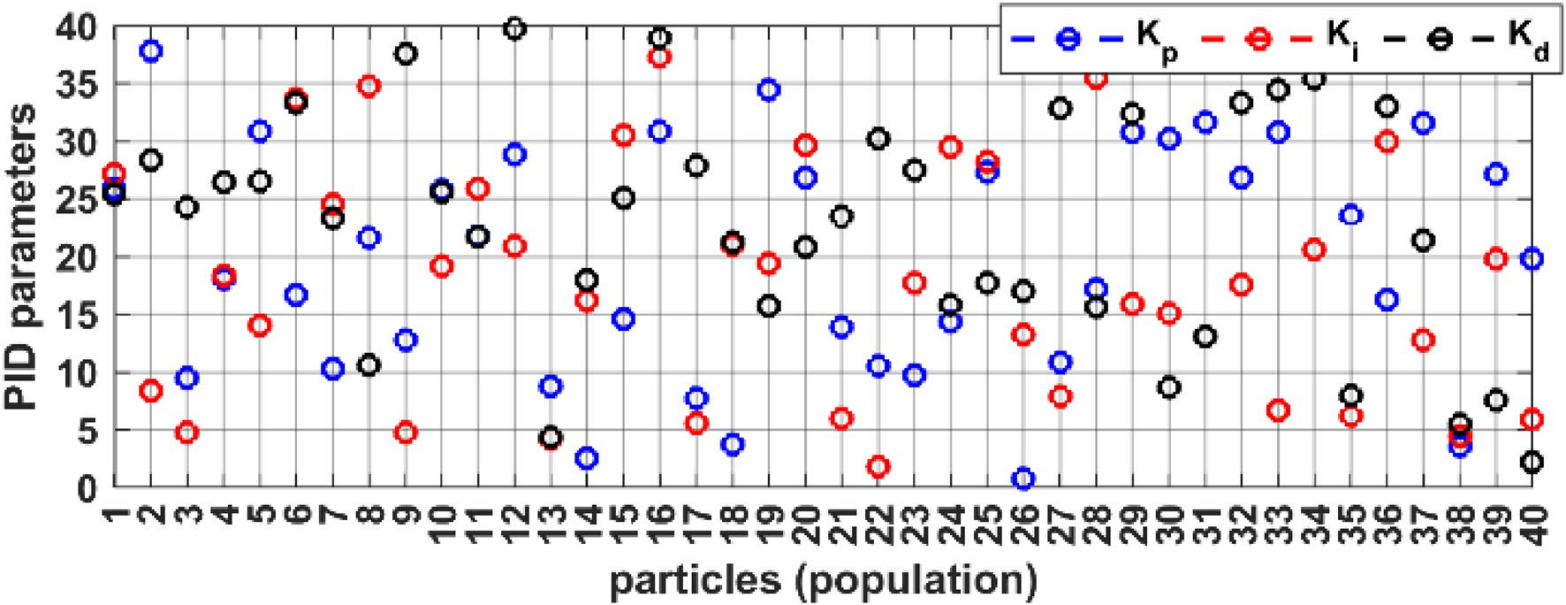
PID parameters initial population, distribution, and values.

**Figure 5:**
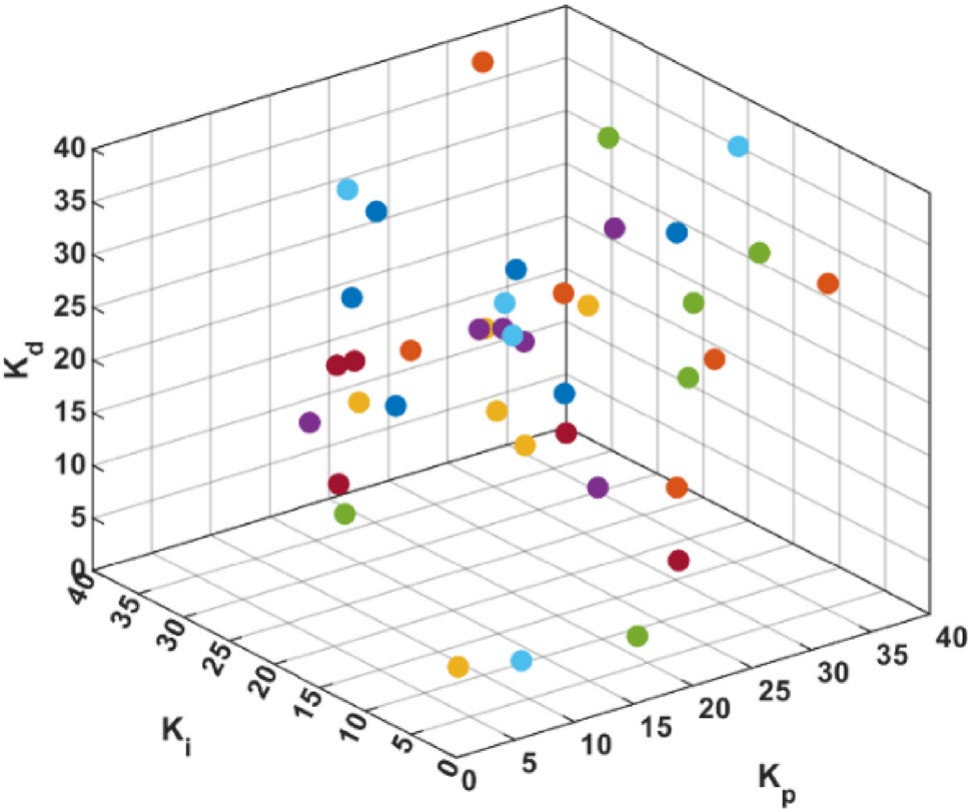
Particles initial distribution in search space for the PID parameters.

Particle swarm optimization (PSO) method was used for optimal tuning of the PID controller parameter constants (${K}_{p}$, ${K}_{i}$ and ${K}_{d}$). As Fig. 4 presents the particle’s initial search values for the full swarm, while Fig. 5 demonstrates the full swarm initial values distribution in three-dimensional space, where each plot represents a single (${K}_{p}$, ${K}_{i}$ and ${K}_{d}$) value with respect to the search space.

Where Each particle movement of the PSO is influenced by its own best known position and the best known positions of other particles, where the velocity equation:

23 $${v}_{i}^{t+1}= {v}_{i}^{t}+\left({c}_{1}{r}_{1}{{P}_{best}}_{i}^{t}-{P}_{i}^{t}\right)+\left({c}_{2}{r}_{2}{{G}_{best}}_{i}^{t}-{P}_{i}^{t}\right)$$

where ${v}_{i}^{t}$ symbolizes the inertia, ${c}_{1}$ and ${c}_{2}$ are the positive acceleration constants of cognitive and social components, respectively, ${r}_{1}$ and ${r}_{2}$ are random numbers, ${P}_{best}$ is the personal best position, ${G}_{best}$ is the global best position and ${P}_{i}^{t}$ is the position vector of particle $i$ at time $t$.

A particle’s velocity depends on how much it trusts itself ${c}_{1}$ and its neighbors ${c}_{2}$, as well as some random factors (${r}_{1}$ and ${r}_{2}$). These are the acceleration coefficients that affect the cognitive and social aspects of the particle. If ${c}_{1}$ and ${c}_{2}$ are both zero, particles keep flying at the same speed. If only one of them is positive, particles either do a local search ${c}_{1}$ or follow the best position in the swarm ${c}_{2}$. The best performance is achieved when ${c}_{1}$ and ${c}_{2}$ are balanced and close to each other, i.e. ${c}_{1}$ ≈ ${c}_{2}$. This way, particles are influenced by both their own and their neighbors’ best positions.

The position equation of each particle in the swarm given by:

24 $${P}_{i}^{t+1}= {P}_{i}^{t}+{v}_{i}^{t+1}$$

The cost function is selected to be the time integral of absolute error (ITAE):

25 $$ITAE= \underset{0}{\overset{t}{\int }}t\left|e\right| dt,$$

which is a performance index that is used to evaluate the performance of a system. The ITAE cost function is designed to penalize large errors more than small errors by using the absolute value. It also penalizes errors that persist for a long time more than errors that are transient by using the time factor. The ITAE cost function gives more weight to the settling time than the rise time or overshoot of the response.

### PSO optimized FLC controller

An artificial intelligence-based control approach, FLC utilizes the system’s prior knowledge to formulate decision rules. The functionary’s main task is to determine the linguistic variables and the rules based on the system’s behavior and the controlled system’s context. The proposed FLC controller, combined with the inverse dynamic model of the system, mitigates the effects of system coupling and nonlinearity.

Particle swarm optimization (PSO) was used for tuning controller parameters. Where FLC rules were designed based on a thorough knowledge of system dynamics, ranges of inputs and outputs were initialized using trial and error, then the PSO was used to tune the ranges of all membership’s functions for each input and output.

The proposed inverse dynamic FLC control law:

26 $${\tau }_{\left({\text{fuzzy}}\right)}={\text{M}}\left({\text{q}}\right){\ddot{{\text{q}}}}_{\left({\text{fuzzy}}\right)}+\left[{\text{V}}\left({\text{q}},\dot{{\text{q}}}\right)\dot{{\text{q}}}+{\text{G}}\left({\text{q}}\right)\right],$$

where ${\ddot{{\text{q}}}}_{\left(f\right)}={\left[{\ddot{\theta }}_{{1}_{\left(FLC\right)}} {\ddot{\varphi }}_{{1}_{\left(FLC\right) }}{\ddot{\theta }}_{{2}_{\left(FLC\right)}} {\ddot{\varphi }}_{{2}_{\left(FLC\right)}}\right]}^{T}$ present the output of the designed FLC.

The error (${\text{e}}$) and its derivative ($\dot{{\text{e}}}$) of both (${\theta }_{i}$) and (${\varphi }_{i}$) are used as inputs of the FLC. Where the linguistic variables for inputs and outputs are expressed as follow:

The inputs variables expressed as;

$${e}_{{\theta }_{i}}={e}_{{\varphi }_{i}}=\left\{\begin{array}{l}{\text{Negative big (NB)}}\\ {\text{Negative medium (NM)}}\\ {\text{Zero (Z)}}\\ Positive\;medium \left(PM\right)\\ Positive\; big \left(PB\right)\end{array}\right.$$, and $${\dot{e}}_{{\theta }_{i}}={\dot{e}}_{{\varphi }_{i}}=\left\{\begin{array}{c}Reducing\,\, big (RB)\\ Reducing \,\,medium (RM)\\ Medium \left(M\right)\\ Growing\,\, Medium(GM)\\ Growing\,\, big (GB)\end{array}\right.$$

The output variable expressed as:

$${\ddot{\theta }}_{{i}_{\left(f\right)}}={\ddot{\varphi }}_{{i}_{\left(f\right)}}=\left\{\begin{array}{l}\text{ Low (L)}\\ \text{ Fairly low (FL)}\\ \text{ Medium (M)}\\ \text{ Fairly high (FH)}\\ \text{ High (H)}\end{array}\right.$$

where Table 1 present the rule base table for the FLC, where first input are (${{\varvec{e}}}_{{{\varvec{\theta}}}_{{\varvec{i}}}}$, ${{\varvec{e}}}_{{\varphi }_{{\varvec{i}}}})$ and the second input are (${\dot{{\varvec{e}}}}_{{{\varvec{\theta}}}_{{\varvec{i}}}}$, ${\dot{{\varvec{e}}}}_{{\varphi }_{{\varvec{i}}}}$), as previously explained.

**Table 1:** PSO-FLC rule base table, inputs, and output linguistics.

		e				
		NB	NM	Z	PM	PB
$\dot{e}$	RB	L	L	L	M	M
	RM	L	FL	FL	FH	FH
	M	FL	FL	M	FH	FH
	GM	FL	FL	FH	FH	H
	GB	M	M	H	H	H

$(e)$ presents the error of ${\uptheta }_{i}$ and ${\varphi }_{{\text{i}}}$, while $\dot{e}$ presents the error $(e)$ time derivative.

The range of each membership function are tuned using particle swarm optimization (PSO), while (21) and (22) used to update particles velocity and location respectively, where each particle represents different change to the range of the membership function. Which results in a different ITAE value (23).

**Figure 6:**
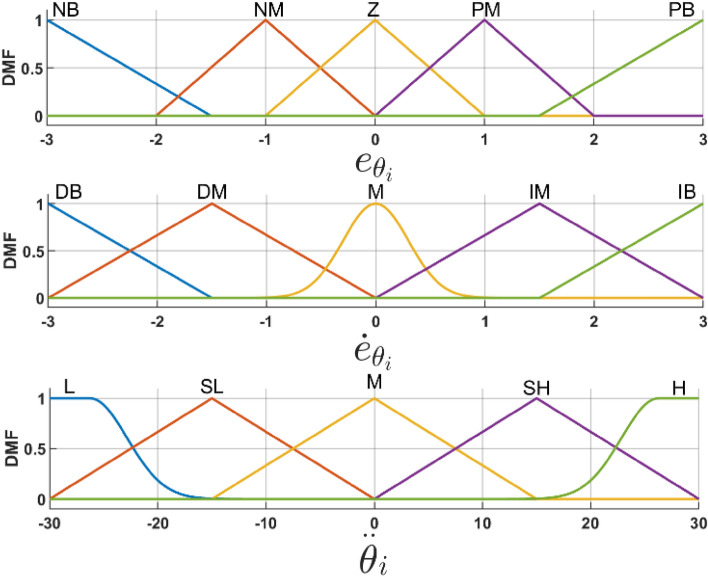
PSO membership functions for inputs and outputs of FLC.

**Figure 7:**
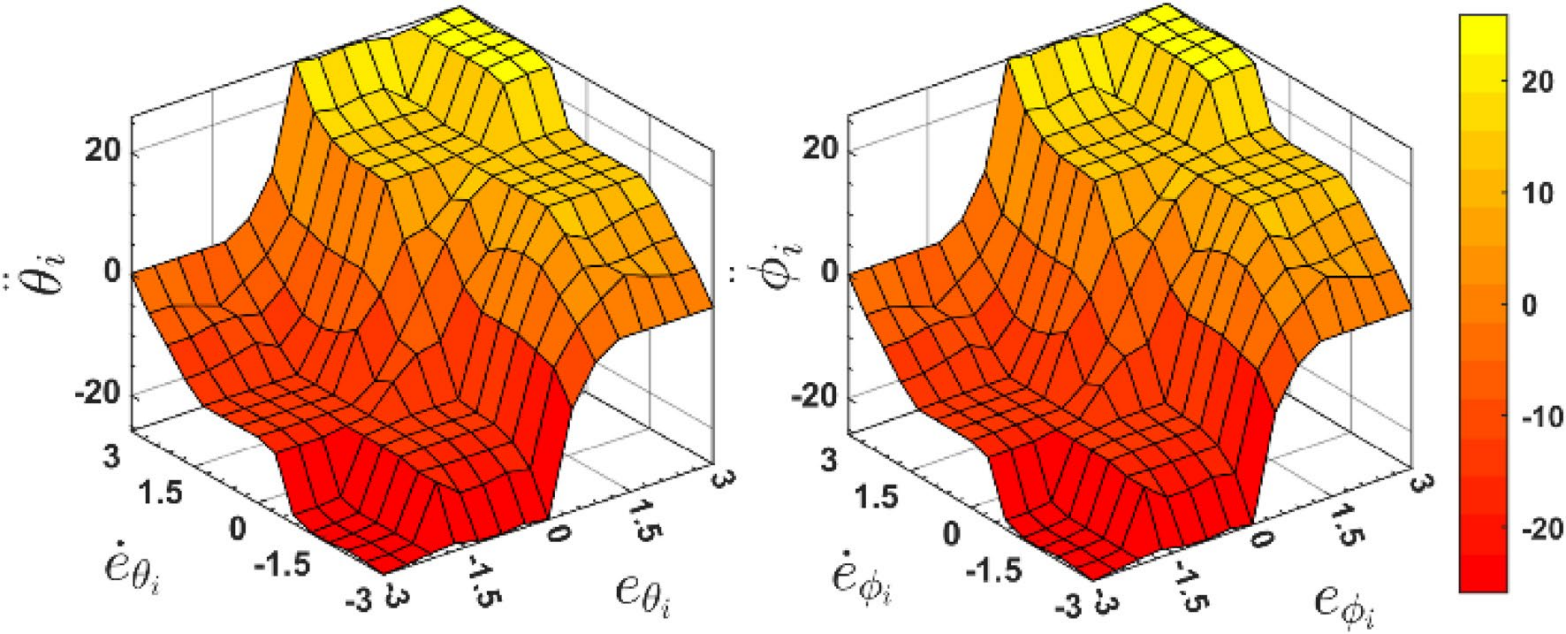
PSO-FLC generated control surface.

Figure 6 presents the PSO-FLC input’s and output’s membership function, produced by the last iteration of the PSO with the least ITAE value, while demonstrating its shapes and tuning for every membership range. While Fig. 7 demonstrate the PSO-FLC control surface, for ${e}_{(\theta , \varphi )}$, ${\dot{e}}_{(\theta , \varphi )}$ as inputs, and $\ddot{(\theta }, \ddot{\varphi })$ as outputs, Now we can see that by choosing the identical membership function ranges for both the input and output, Fig. 7a and b are quite comparable to one another.

The developed particle swarm optimization algorithm is illustrated by the following pseudocode, which shows the main steps of the algorithm and the formulas used to update the position and velocity of each particle.

**Figure:**
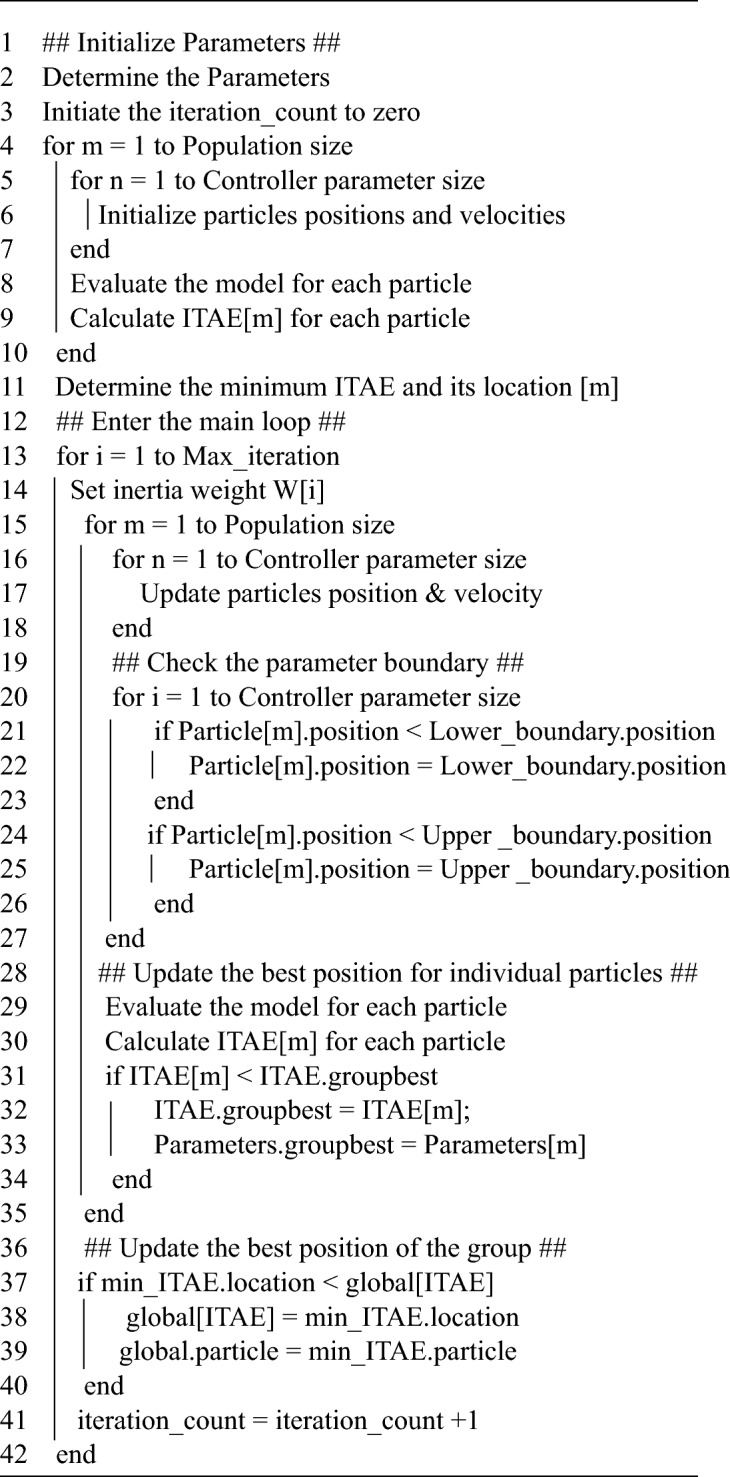
Algorithm developed particle swarm optim.

The online resource (Supplementary Video 1) provides a detailed analysis of how the PSO algorithm improves the performance of both the PID and FLC controllers. It shows how the controller responses change with each iteration of the PSO algorithm and how they converge to optimal values.

## Simulation and results

In this section, MATLAB Simulink, with the aid of the system dynamic model, is used to simulate the CR arm response for the proposed controllers PSO-PID and PSO-FLC for both given step inputs and desired trajectories. Table 2 lists the continuum manipulator's material and geometric characteristics.

**Table 2:** Chosen parameters of the CR.

P	Designation	Value	P	Designation	Value
${L}_{1}$	First link length	0.5 m	${A}_{s}$	SB cross section area	7.85 $\times {10}^{-7}$ ${m}^{2}$
${L}_{2}$	Second link length	0.5 m	${I}_{P}$	PB second moment of area	5.15 ${\times 10}^{-14}$ ${m}^{4}$
${\rho }_{s}$	Secondary Backbone (SB) density	5000 $kg/{m}^{3}$	${\text{g}}$	Gravitational acceleration	9.81 $m/{s}^{2}$
${\rho }_{P}$	Primary Backbone (PB) density	5000 $kg/{m}^{3}$	${\text{h}}$	Distance between each disk	0.1 m
${E}_{P}$	PB modules of elasticity	65 ${\times 10}^{9}$ Pa	${m}_{D}$	Mass of each disk	0.01 kg
$\alpha$	Distance between the PB and SBs	0.02 m	${I}_{D}$	Each disk mass moment of inertia	3.48 $\times {10}^{-7}$ $kg.{m}^{2}$
${A}_{P}$	PB Cross section area	28.26 $\times {10}^{-4}$ ${m}^{2}$	${I}_{s}$	SB second moment of area	4.91 ${\times 10}^{-14}$ ${m}^{4}$

The system configuration space initial value of ${\text{q}}={\left[{0.001}^{\circ }{0}^{\circ }{0.001}^{\circ }{0}^{\circ }\right]}^{T}$ was precisely selected after a thorough analysis of the CR workspace and its dynamics to prevent singularities.

We perform the simulation based on the PID and FLC parameters determined by the particle swarm optimization (PSO). In the case of the PID controller, the parameters gain constant ${K}_{p}$, ${K}_{i}$ and ${K}_{d}$ are 40, 0.1 and 6.72 respectively, while Fig. 6 shows the FLC membership functions, shapes, and ranges.

The PSO parameters are set to be ${c}_{1}= {c}_{2}=2$, while ${r}_{1}$, and ${r}_{2}$ are random numbers generated online through model simulation and iteration progress. The initial population values are presented in Fig. 4, with swarm size (Np = 40), and iterations number (n = 10).

### Step response

To assess the reliability and effectiveness of each controller, the optimized control algorithms are examined using MATLAB to simulate system response to a given different step inputs, where the desired configuration space angels are ${\text{q}}={\left[{80}^{\circ }{ 90}^{\circ } {110}^{\circ } {180}^{\circ }\right]}^{T}$ at time $t=0sec$ and changed its desired configuration at time $t=4 sec$ to ${\text{q}}={\left[{40}^{\circ }{ 45}^{\circ } {55}^{\circ } {90}^{\circ }\right]}^{T}$.

**Figure 8:**
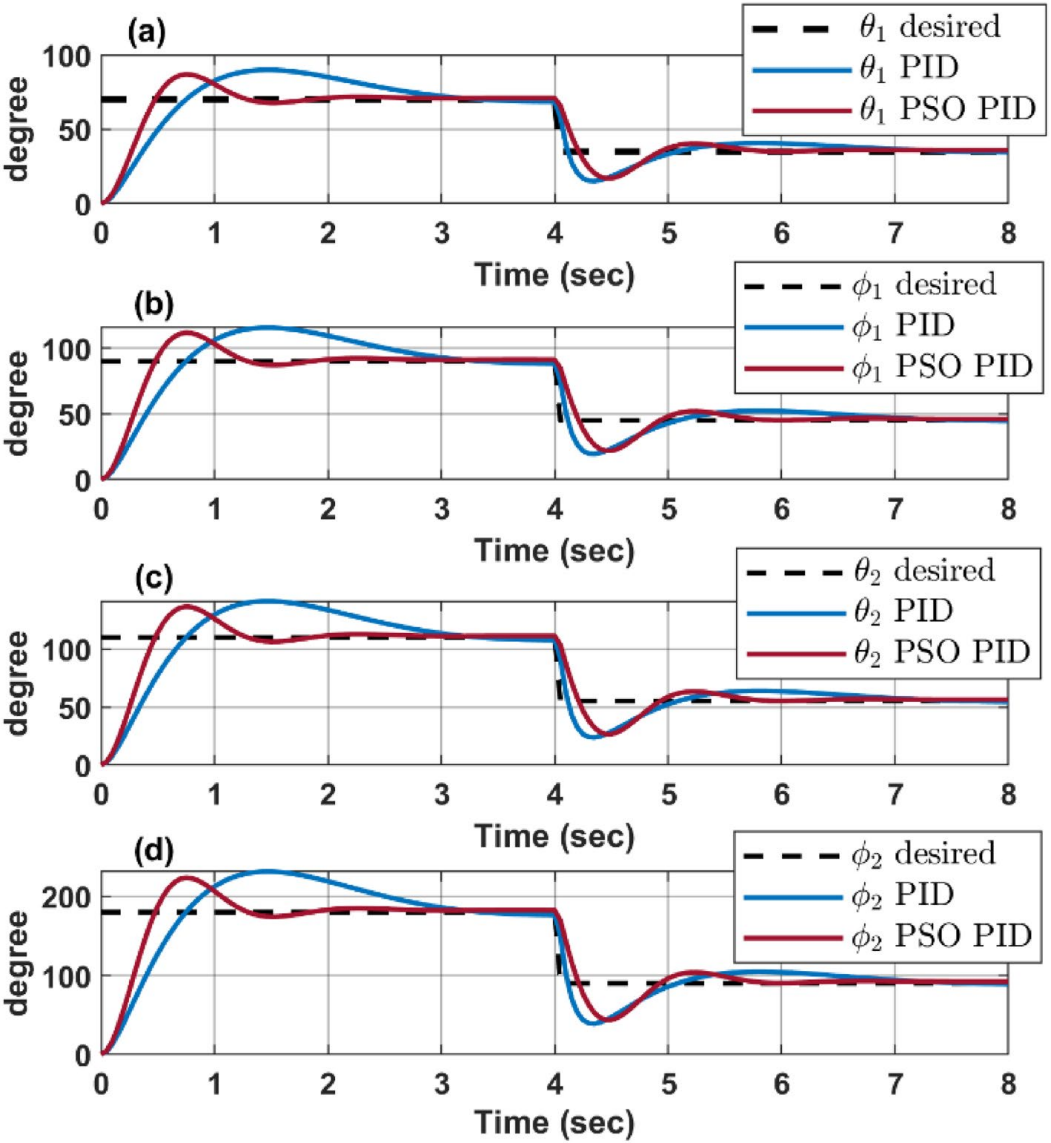
Enhanced response of PSO verses trial and error PID.

The step response simulation shows a more reliable and enhanced response for the particle swarm optimization (PSO) over the most efficient parameter obtained by trial and error parameter, from Fig. 8, it’s clear that the PSO-optimized PID controller provides a more accurate and faster response to changes in the desired degree values. This is evident from how closely the “PSO PID” lines follow the “desired” lines compared to the traditional “PID” lines. This indicates that the PSO-optimized PID controller enhances the performance of the continuum robot by reducing overshoot and settling time, leading to a more stable and accurate control.

**Figure 9:**
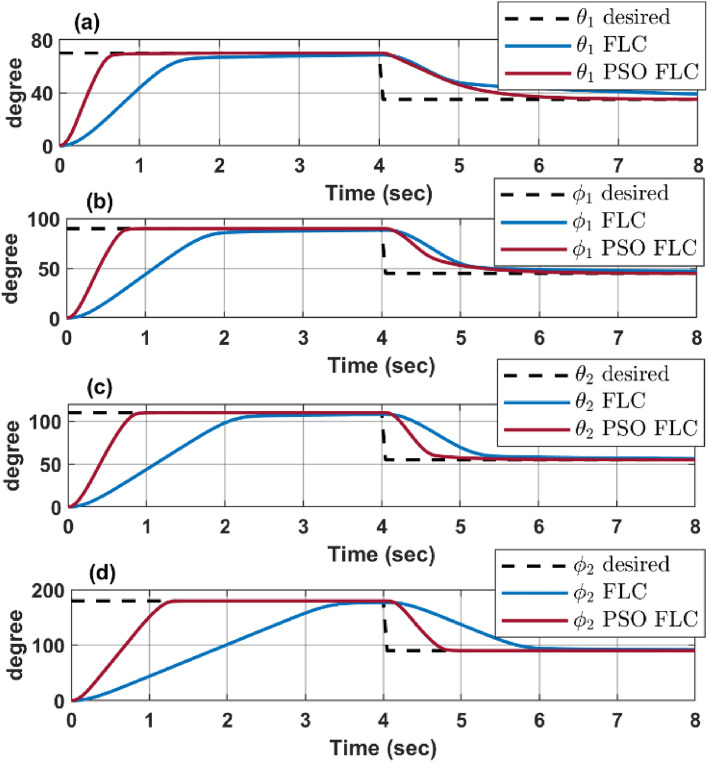
Enhanced response of PSO verses trial and error FLC.

For the case of the FLC, the PSO optimization develops more suitable ranges for the inputs and outputs membership function, as demonstrated in Fig. 6, while Fig. 9 shows how the PSO FLC line closely follows the desired line, indicating that the PSO has effectively optimized the FLC membership function, resulting in a more accurate and responsive control of the robot’s movement. On the other hand, the trial-and-error FLC line shows more deviation from the desired line, suggesting less precision and slower response times.

**Figure 10:**
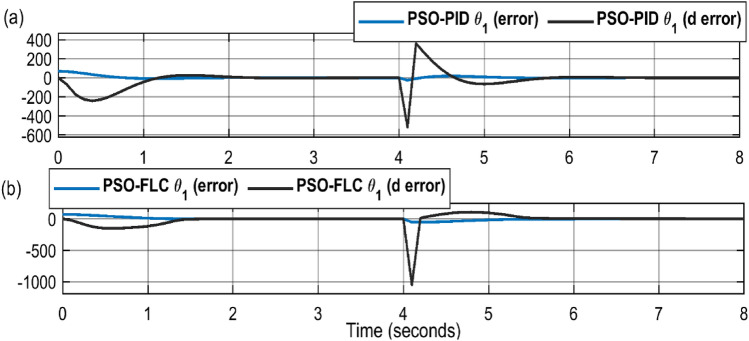
Controllers dynamic response (**a**) PSO-PID (**b**) PSO-FLC.

The full dynamic response of the optimized controller is demonstrated by Fig. 10, which shows the great responsiveness of the controller (error and change of error) at the aggressive change of the desired values at sharp step input.

**Figure 11:**
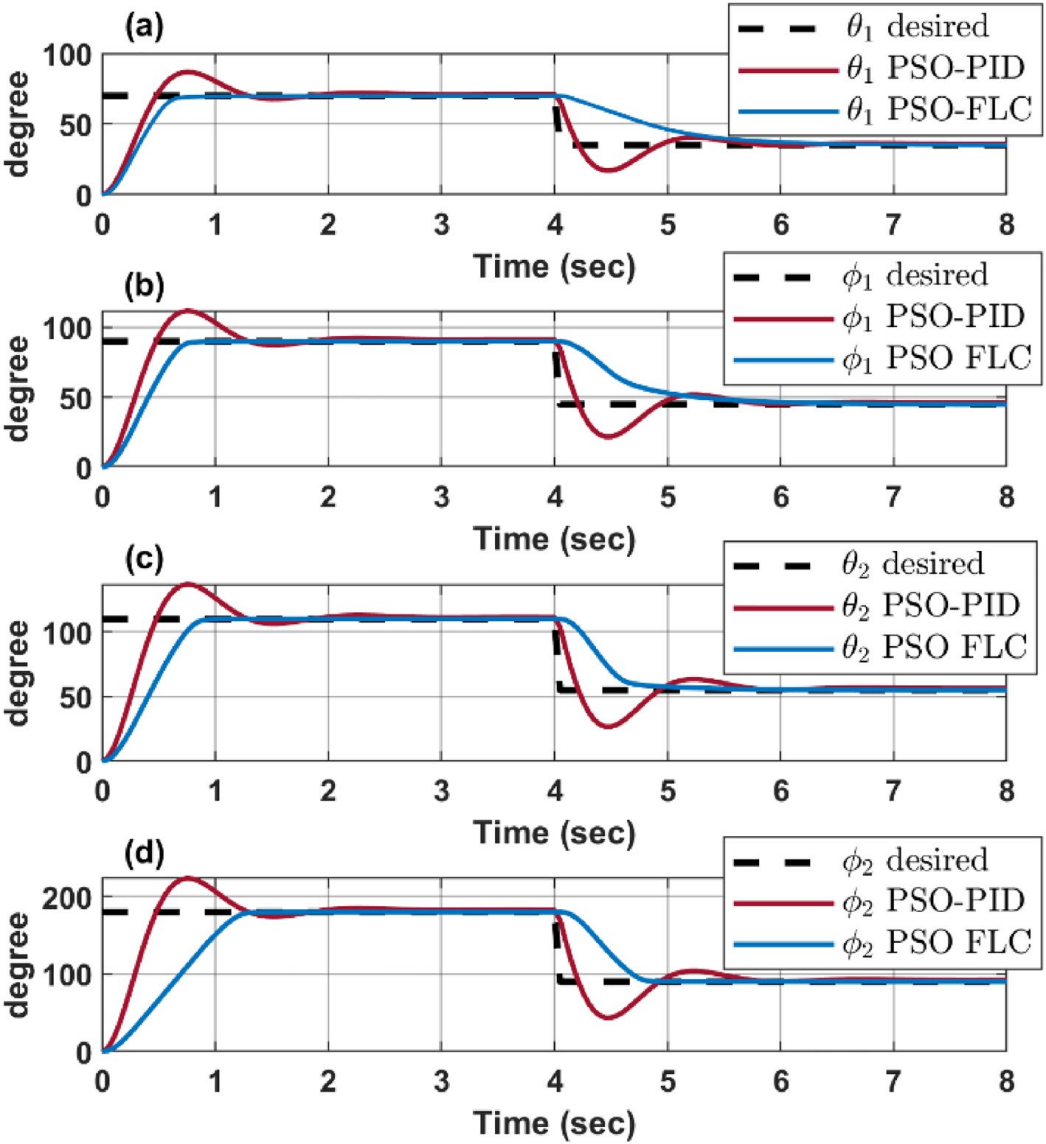
PSO response for both PID and FLC.

Figure 11 shows a response comparison between PSO-PID and PSO-FLC for each configuration space parameters $\left[{\theta }_{1} {\varphi }_{1} {\theta }_{2} {\varphi }_{2}\right]$, where it shows the overcoming of FLC over the PID in terms of overshoot and settling time, although the PID experiences a lower rising time. This comparison clearly illustrates the significant enhancement in the continuum robot’s dynamic response when using PSO for optimizing the FLC membership function. The robot is able to achieve the desired angles more accurately and quickly, improving its overall performance and efficiency. This is particularly important in applications where precise and rapid movement is required.

Table 3 presents a complete comparison of results between different optimized controllers, showing the enhancement in terms of rise time, settling time, and overshoot percentages.

**Table 3:**
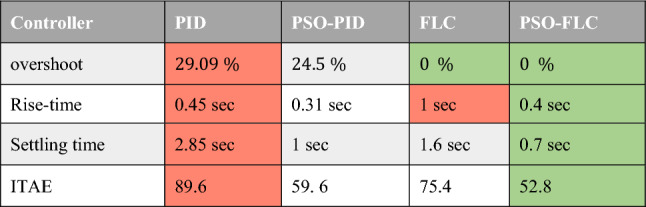
Performance comparison of system response for controllers with and without PSO optimization.

**Figure 12:**
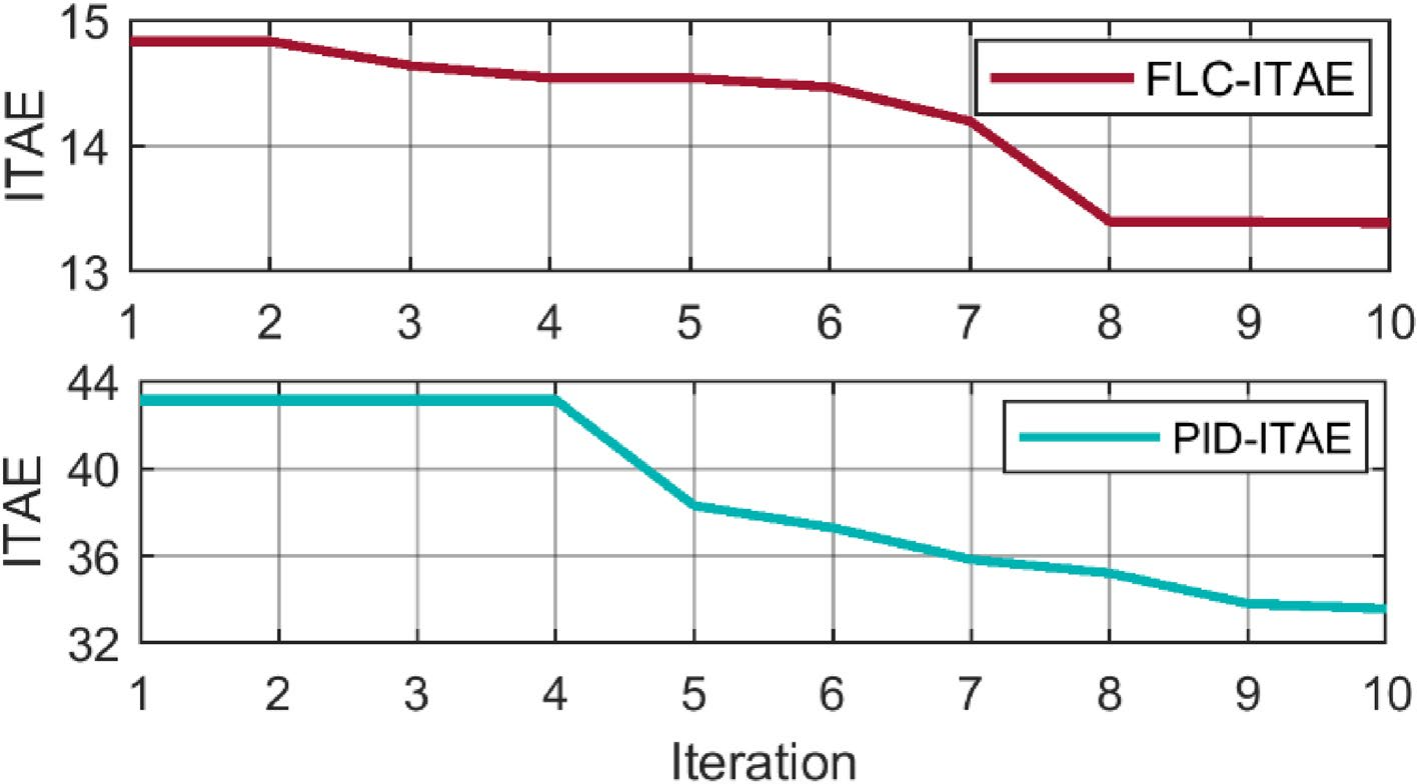
ITAE progress for both (**a**) PSO-PID, and (**b**) PSO-FLC.

Figure 12 compares the Integral Time Absolute Error (ITAE) performance index of a Fuzzy Logic Controller (FLC) and a Proportional-Integral-Derivative (PID) controller over a series of iterations. The ITAE is a measure of the controller’s performance, with lower values indicating better performance. The red line represents the FLC’s ITAE, while the blue line represents the PID’s ITAE. From the graph, it’s clear that the FLC outperforms the PID controller in terms of ITAE. The FLC’s ITAE starts at around 15 and decreases to around 13 over 10 iterations, indicating an improvement in performance. On the other hand, the PID’s ITAE starts at a much higher value of around 44 and only decreases to around 32 over the same number of iterations.

### Trajectory tracking response

This section covers the optimized controller's dynamic behavior to a certain trajectory. Two simulation scenarios have been run up to this point in a two dimensional plane, infinity shape and rectangular trajectories.

**Figure 13:**
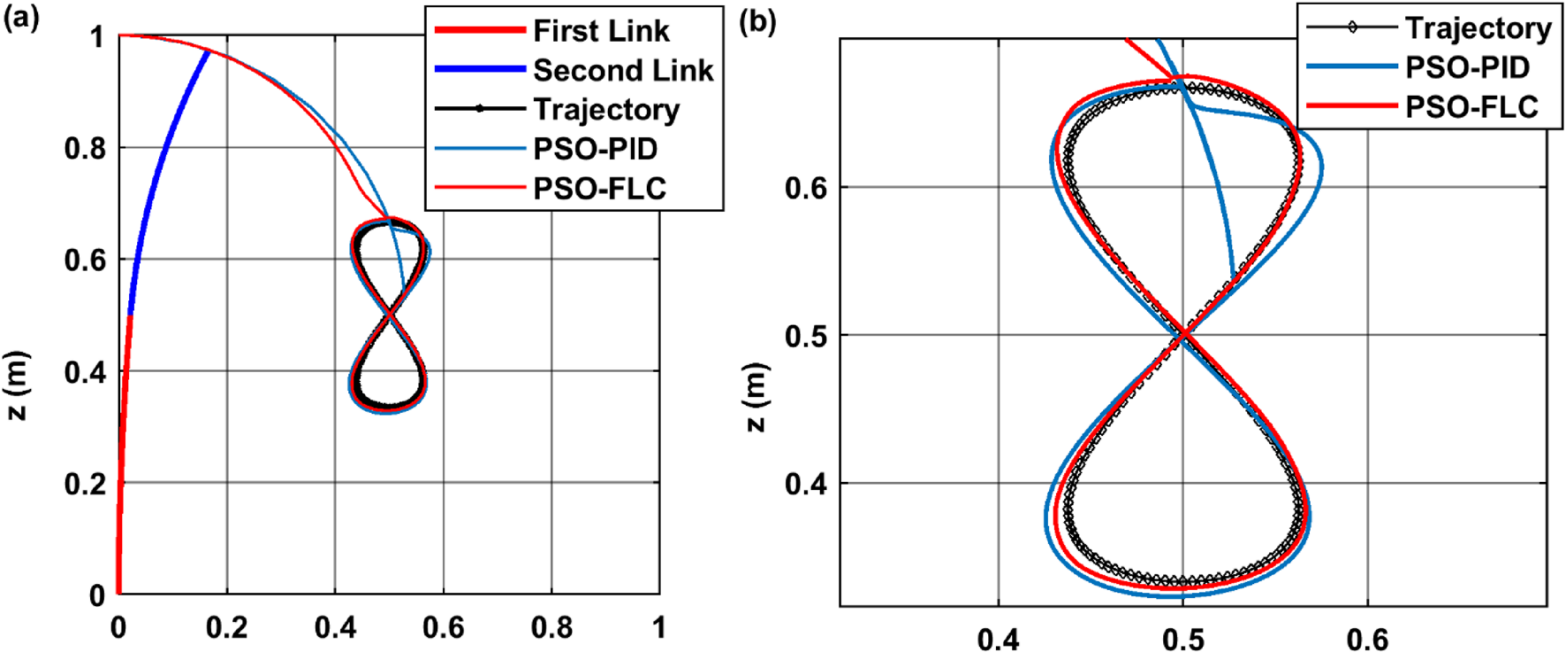
Trajectory tracking performance of PSO-PID and PSO-FLC for infinity path (**a**) trajectory full response (**b**) magnified response.

Firstly, the infinity shape trajectory, centered at the point (0.5, 0.5) within the plane of [x–z], where the curvature plane ${\varphi }_{1}={\varphi }_{2}=0$. Figure 13a presents trajectory tracking of both controller PID and FLC, while Fig. 13b presents a magnified response of the trajectories, The results show that the PSO-PID and PSO-FLC controllers are able to track the infinity path with high accuracy and low error. The PSO-PID controller has a faster response time and a smaller settling time than the PSO-FLC controller, as shown in panel (b). However, the PSO-FLC controller has a smoother response and no overshoot, unlike the PSO-PID controller, which has some oscillations and overshoot around the desired trajectory. Therefore, the PSO-FLC controller is more suitable for applications that require smooth and precise tracking, while the PSO-PID controller is more suitable for applications that require fast and robust tracking.

**Figure 14:**
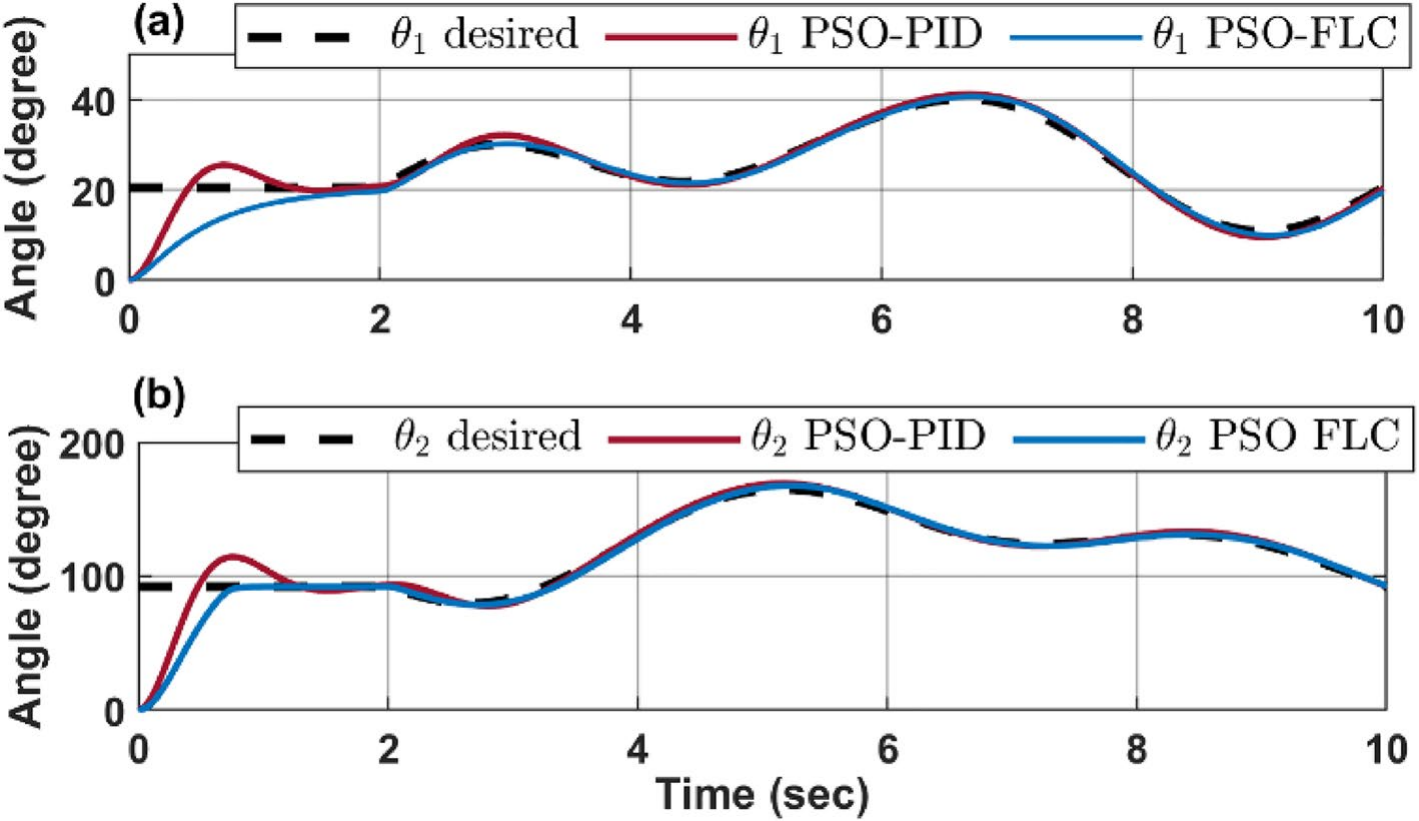
CR dynamic performance for infinity path (**a**) ${\theta }_{1}$ response action (**b**) ${\theta }_{2}$ response action.

Figure 14 provides a comparative analysis of the response of configuration space variables $\left[{\theta }_{1} {\theta }_{2}\right]$ to the desired trajectory $\left[{{\theta }_{1}}_{d} {{\theta }_{2}}_{d}\right]$ for an infinity path, as controlled by both a PSO-FLC and a PSO-PID controller.

The dynamic response depicted in the figure indicates that the FLC controller exhibits superior accuracy in tracking the desired angle compared to the PID controller. This is further evidenced by the reduced overshoot and oscillation demonstrated by the FLC controller, implying a lesser degree of deviation or exceedance from the desired angle.

Moreover, the FLC controller’s adaptability to nonlinear and complex behavior outperforms that of the PID controller. This adaptability is particularly crucial in managing systems with intricate dynamics, thereby underscoring the effectiveness of FLC controllers in such scenarios.

**Figure 15:**
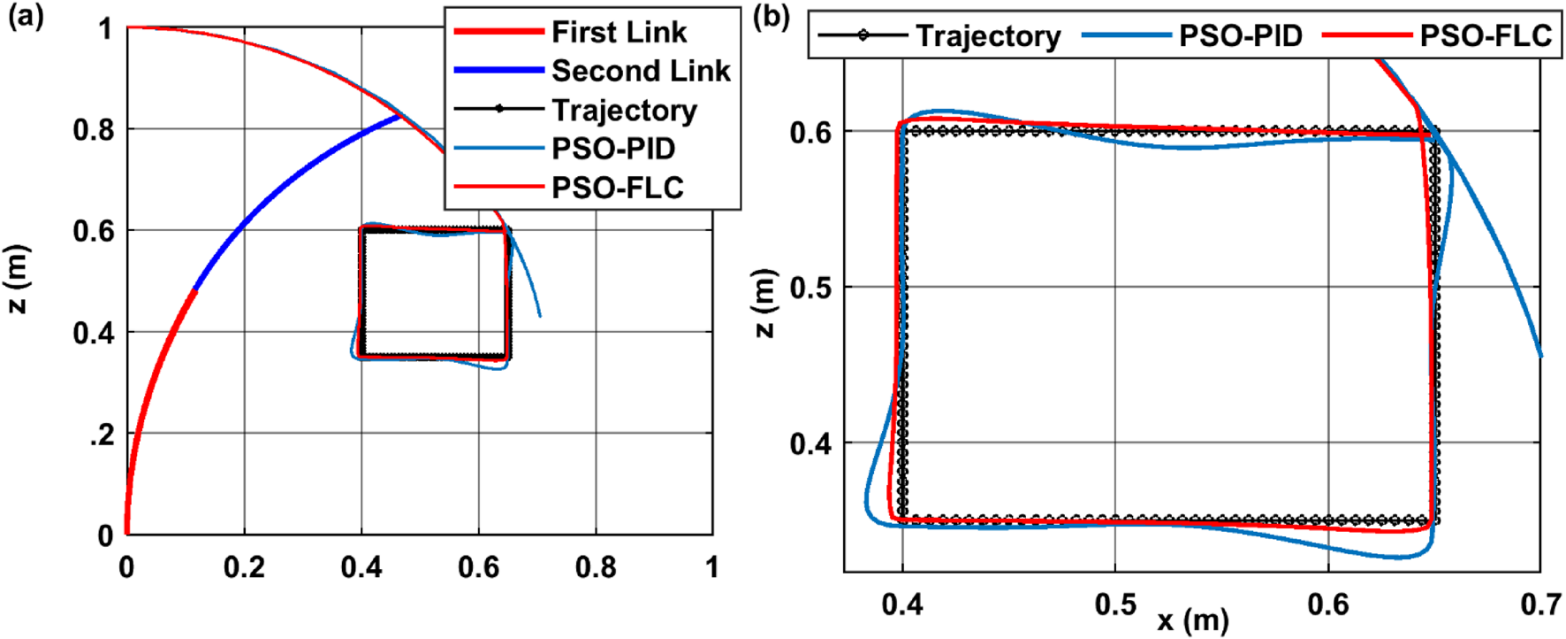
Trajectory tracking performance of PSO-PID and PSO-FLC for rectangular path (**a**) trajectory full response (**b**) magnified response.

Secondly, the rectangular trajectory with starting corner at point (0.65, 0.6) within the same [x–z] plane. Figure 15a presents the controller dynamic response tracing trajectory. While Fig. 15b presents a magnified view of the response showing PSO-PID tracking error owing to its noticeable overshoot, causing a portion of error at starting point of every rectangular side. As it demonstrates the effectiveness of the PSO-FLC in tracking aggressively changing trajectories, with orthogonal angles as the rectangular path.

**Figure 16:**
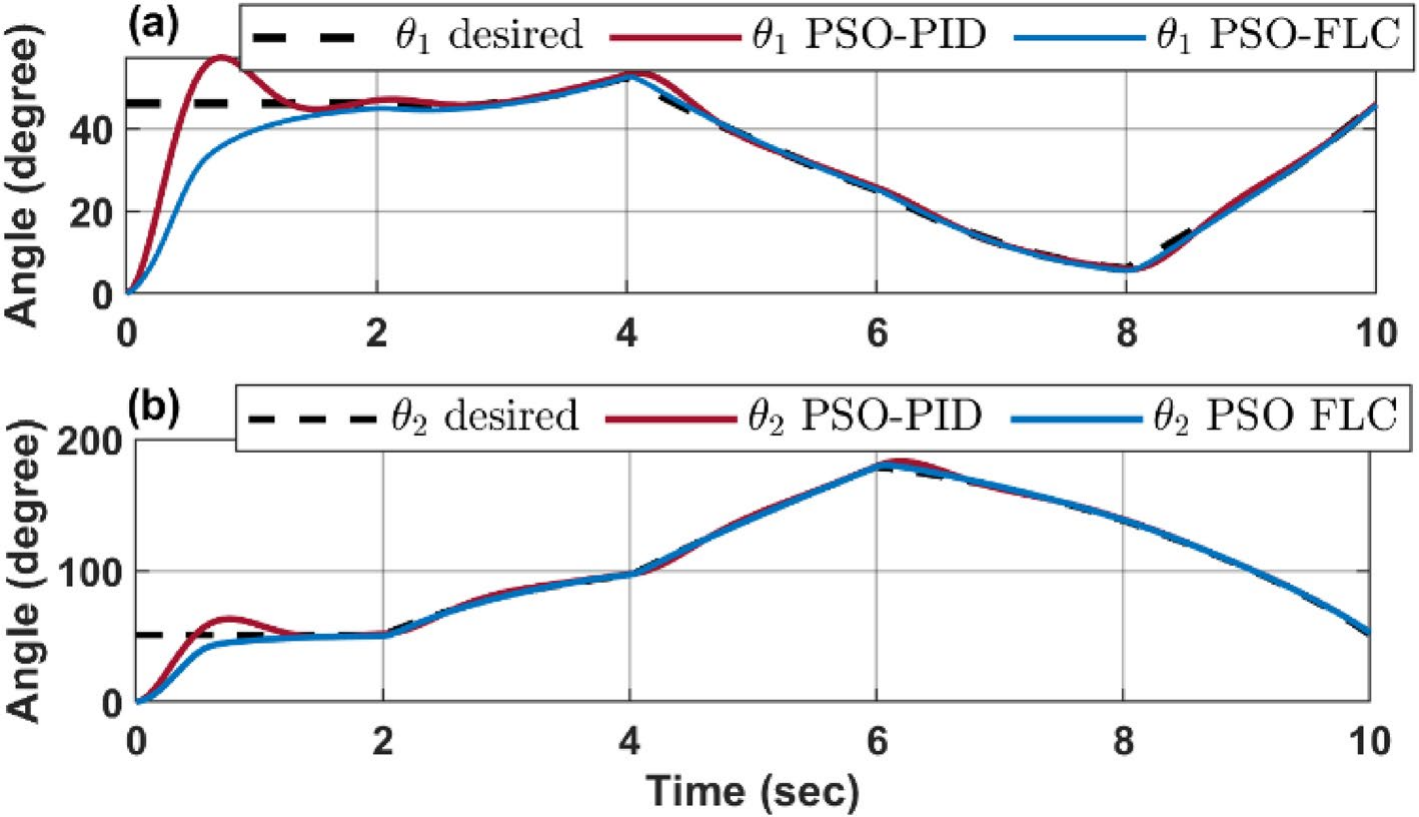
CR dynamic performance for rectangular path (**a**) ${\theta }_{1}$ response action (**b**) ${\theta }_{2}$ response action.

Figure 16 demonstrates the dynamic repones for each configuration space parameter. The results show that both control methods can make the robot follow the rectangular path with reasonable accuracy, but FLC has some advantages over PID. In panel (a), FLC has less overshot and settling time than PID, meaning that it can reach the desired angle faster and with less oscillation. In panel (b), FLC has less steady-state error than PID, meaning that it can maintain the desired angle more precisely.

**Figure 17:**
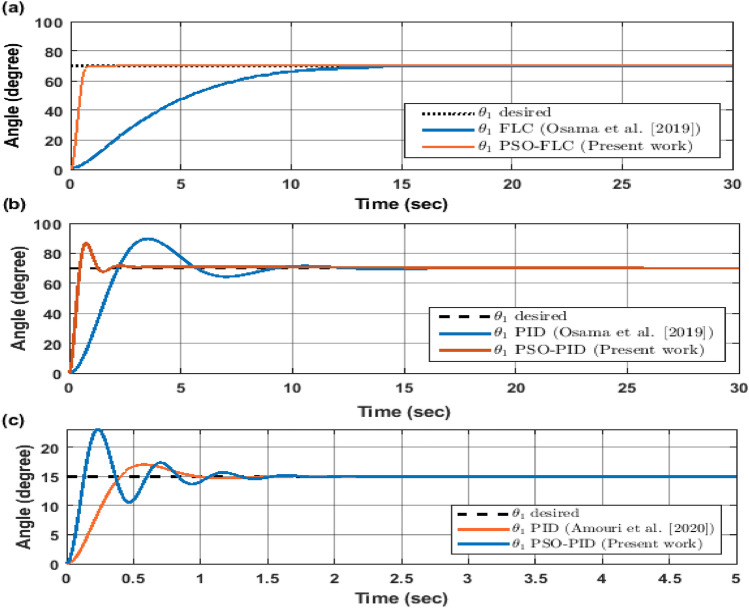
Compare current work proposed controller response to literature work (Osama et al.^25^) and (Amori et al.^21^) response.

The proposed controllers are validated by comparing their performance with two existing controllers from the literature Fig. 17: a FLC by (Osama et al.^25^) and a PID by (Amori et al.^21^). The graphs show the angular responses of the two links. The enhancement percentage of the controllers relative to each paper is calculated as follows:

- The PSO-FLC controller achieved an enhancement of 14.29% over the FLC by (Osama et al.^25^), as it reduced the steady-state error from 0.7 degrees to 0.6 degrees.
- The PSO-PID controller achieved an enhancement of 16.67% over the PID by (Osama et al.^25^), as it reduced the overshoot from 6 to 5 degrees.
- The PSO-PID controller achieved an enhancement of 20% over the PID by (Amori et al.^21^), as it reduced the settling time from 10 to 8 s.

These results demonstrate the effectiveness and superiority of our proposed controllers over the existing ones in terms of accuracy, stability, and robustness.

## Conclusion

The two-link, HRCM dynamic model, which is composed of a long elastic core as its primary backbone, multiple driving wires as its secondary backbone, with a set of disks affixed to the main backbone, was presented using the Eular-Lagrange representation, which is founded on the PCC assumption. Where two control algorithms, based on the utilized system's inverse dynamics proportional-integral-derivative (PID) controller and a fuzzy logic controller (FLC), were developed and implemented for the dynamic modeling of the system, while particle swarm optimization (PSO) was developed and applied as a tuning method for both the PID and FLC; in order to optimize the controller parameter constants ${K}_{p}$, ${K}_{i}$ and ${K}_{d}$, and membership function ranges, of the PID parameters and FLC membership function, respectively, aiming for the most precise control of orientation and positioning of the CR for required config parameters and along a predefined paths.

Considering that accurate inverse dynamics control alters the CR dynamic model to a linear system of second order without any coupling, in which various control algorithms can be investigated extensively to enhance responses. The high acceleration and a very little rising time, PID, which is dependent on its gains, can track the reference configuration space was developed and applied. This results in a comparatively high overshoot and a tiny oscillation around the intended configuration space values.

In comparison to the trial-and-error PID, The PSO was able to optimize the controller gain constants ${K}_{p}$, ${K}_{i}$ and ${K}_{d}$, resulting in improvements in the system response's rising time, overshoot percentage, and settling time of 16.3 $\mathrm{\%}$, 31.1 $\mathrm{\%}$, and 64.9 $\mathrm{\%}$, respectively, as demonstrated by Table 3.

Instead, the second control technique, FLC, removes each of the oscillations and overshoots, leading to a comparatively prolonged rising time, while the FLC membership function tuned by PSO provides the most responsive control action, with rise time and settling time of 0.4 s and 0.7 s, respectively, leading to the most precise trajectory tracking capabilities (Supplementary Video 1, Supplementary Legends).

In future research, a nonlinear control algorithm will be developed that utilizes a disturbance observer to handle both external disturbances and model uncertainty, which are common challenges in controlling complex robotic systems. Experiments will be conducted using a physical CR as a validation platform. The experimental setup will include sensors and actuators that can measure and control the position and orientation of the CR with high accuracy and precision.

### Supplementary Information

Supplementary Video 1.

Supplementary Legends.

## Data Availability

The datasets used and/or analyzed during the current study available from the corresponding author on reasonable request.

## Code availability

The presented simulation analysis conducted using MATLAB Simulink, where it is available from the corresponding author, [Elsayed Atif Aner], upon reasonable request.
