# Supplementary material for: Performance evaluation of PSO-PID and PSO-FLC for continuum robot’s developed modeling and control
Source: Sci Rep. 2024 Jan 6;14:733. doi: 10.1038/s41598-023-50551-0 (PMC10771498; doi:10.1038/s41598-023-50551-0)
Supplement: Supplementary file 2 — Supplementary Legends. [file 41598_2023_50551_MOESM2_ESM.pdf]

Supplementary Video Legends

In this video, we present a Particle Swarm Optimization (PSO) algorithm that we applied to the inverse dynamic PID and FLC controllers that we developed for a continuum robot. The video shows how the PSO algorithm evolves through the iterations, starting from the initial distribution of the particles in the search space, and displaying the parameter values, the ITAE value, and the system response for the best ITAE particle in each iteration. The video also shows an animated 3D motion of the continuum robot as it tracks different desired trajectories, and graphically demonstrates the system dynamic response. Finally, the video summarizes the main results in a table.
